# φYeO3-12 phage tail fiber Gp17 as a promising high specific tool for recognition of *Yersinia enterocolitica* pathogenic serotype O:3

**DOI:** 10.1186/s13568-021-01341-2

**Published:** 2022-01-06

**Authors:** Karolina Filik, Bożena Szermer-Olearnik, Joanna Niedziółka-Jönson, Ewa Roźniecka, Jarosław Ciekot, Anna Pyra, Irwin Matyjaszczyk, Mikael Skurnik, Ewa Brzozowska

**Affiliations:** 1 Hirszfeld Institute of Immunology and Experimental Therapy, Polish Academy of Sciences, 12 R. Weigl St, 53114 Wroclaw, Poland; 2 Institute of Physical Chemistry, Polish Academy of Sciences, Kasprzaka 44, 5201-224 Warsaw, Poland; 3 Faculty of Chemistry, University of Wroclaw, 14 F. Joliot-Curie St, 50383 Wroclaw, Poland; 4 Department of Mycology and Genetics, Institute of Genetics and Microbiology, University of Wrocław, 51-148 Wroclaw, Poland; 5 Department of Bacteriology and Immunology, Faculty of Medicine, Human Microbiome Research Program, University of Helsinki, Helsinki, Finland; 6 Division of Clinical Microbiology, Helsinki University Hospital, HUSLAB, Helsinki, Finland

**Keywords:** Phage, Yersiniosis, Tail fiber protein, Phage adhesins, *Yersinia enterocolitica*, Diagnostic, ELISA

## Abstract

Yersiniosis is an infectious zoonotic disease caused by two enteropathogenic species of Gram-negative genus *Yersinia*: *Yersinia enterocolitica* and *Yersinia pseudotuberculosis*. Pigs and other wild and domestic animals are reservoirs for these bacteria. Infection is usually spread to humans by ingestion of contaminated food. Yersiniosis is considered a rare disease, but recent studies indicate that it is overlooked in the diagnostic process therefore the infections with this bacterium are not often identified. Reliable diagnosis of Yersiniosis by culturing is difficult due to the slow growth of the bacteria easily overgrown by other more rapidly growing microbes unless selective growth media is used. Phage adhesins recognizing bacteria in a specific manner can be an excellent diagnostic tool, especially in the diagnosis of pathogens difficult for culturing. In this study, it was shown that Gp17, the tail fiber protein (TFP) of phage φYeO3-12, specifically recognizes only the pathogenic *Yersinia enterocolitica* serotype O:3 (YeO:3) bacteria. The ELISA test used in this work confirmed the specific interaction of this protein with YeO:3 and demonstrated a promising tool for developing the pathogen recognition method based on phage adhesins.

**Supplementary Information:**

The online version contains supplementary material available at 10.1186/s13568-021-01341-2.

## Key points

1. TFP of φYeO3-12 phage was shown to be an excellent tool for YeO:3 detection.
2. It is beneficial to leave MBP in the complex with TFP.
3. The specific interaction of H/MTFP-Gp17 and the pathogenic bacteria was shown in ELISA and TEM.

## Introduction

Yersiniosis is an infection in human-caused by *Yersinia enterocolitica* (Ye) bacteria. Most often the infection is caused by eating raw or undercooked pork but also dairy products contaminated by the bacteria. It was shown that Ye is the third most common enteric pathogen responsible for food poisonings with dairy products. In the United States, Ye causes almost 117,000 illnesses, 640 hospitalizations, and 35 deaths every year. In Europe, 7048 confirmed cases of yersiniosis (caused by *Y. enterocolitica* and *Y. pseudotuberculosis*) were reported by the European Food and Waterborne Diseases and Zoonoses (FWD) Network for 2019 and was the fourth most commonly reported foodborne zoonotic disease in the European Union. Children are infected more often than adults, and the infection is more common in cooler climates. Yersiniosis is typically an enteric disease and signs may include diarrhea, weight loss, severe abdominal pain, dehydration, and bloody feces (Watkins and Fredman, 2020; Triantafillidi et al., 2020). In all countries, the infection rate is most likely much higher since only serious cases are registered, and for many reasons the infections may be overlooked (Wielkoszynski et al., 2018). The bacteria of genus *Yersinia* are Gram-negative coccobacilli that belong to the *Enterobacterales* order, *Yersiniaceae* family. Biochemically and serologically they have been categorized into three species (*Y. enterocolitica, Y. pestis, Y. pseudotuberculosis*), which are responsible for infections in humans. *Y. enterocolitica* isolates, based on their biochemical properties have been divided into six biotypes, and on their antigenic properties, into 70 serotypes (Shoaib et al., 2019). According to pathogenicity and geographical distribution, it is categorized into six distinct groups corresponding to the biotypes defined by their biochemical properties: 1A, 1B, 2, 3, 4, 5. Within these biotypes of Ye are different serotypes and some of them belong to each biota as follows: 1A (O:5; O:6, 30; O:7, 8; O:18; O:46), 1B (O:8; O:4; O:13a, 13b; O:18; O:20; O:21), 2 (O:9; O:5, 27), 3 (O:1, 2, 3; O:5, 27), 4 (O:3) and 5 (O:2,3). Typically, serotypes O:3, O:8, O:9, and O:5, 27 have been associated with virulence and cause most of the infections. In addition, there is a geographical distribution; serotypes O:4, O:8, O:13a/b, O:18, O:20, and O:21 are prevalent in USA, while serotypes O:3 and O:9 dominate in Europe and Japan (Wielkoszynski et al., 2018; Simonova et al., 2007). The serotypes are sometimes described as bio/serotypes such as 4/O:3. 4/O:3 is the most frequently isolated *Y. enterocolitica* in Europe that cause asymptomatic infections in pigs thereby contaminating the pork meat and causing human infections (Batzilla et al., 2011). YeO:3 appears to be an example of a zoonotic pathogen perfectly adapted to infect humans. It can be seen in the genomic variations of YeO:3 that streamline the physiology and metabolism of the bacteria (Schmühl et al., 2019). It is commonly known that the composition and modification of mucin is a critical defense mechanism in the prevention of pathogenic bacteria in the intestine. The amount of *N*-acetyl-d-galactosamine (GalNAc) is nearly twice that of any of the other sugars present in the mucin of the small intestines of pigs. In contrast, *N*-acetyl-d-glucosamine (GlcNAc) is the major amino sugar in human mucin. One of the adaptive features of the YeO:3 bacteria in contrast to the nonpathogenic Ye bacteria, is the ability to uptake GlcNAc and GalNAc as a source of carbon (Schmühl et al., 2019).

Ye remains a challenge for researchers and food handlers due to its ability to grow at refrigeration temperature, low concentrations in samples, morphological similarities with other bacteria and lack of rapid, cost-effective, and accurate detection methods. The recommended method of Ye isolation is carried out by using ISO 10273-2003 (Morka et al., 2018). In this protocol, body fluids (from the peritoneum, wounds, or abscesses) or stools are taken to analysis, and bacteria are inoculated on enrichment peptone sorbitol bile (PSB) broth, irgasan-ticarcillin-potassium chlorate (ITC) broth, and cefsulodin-irgasan-novobiocin (CIN). To isolate presumptive strains of Ye, the ITC broth and CIN agar media are recommended. Colonies of *Yersinia* sp. on CIN agar have the bull’s eye morphology with a red center and colorless translucent rims (Morka et al., 2018). After bacterial colony isolation, the Ye biotypes are identified via a biochemical characterization using commercial systems such as API 20E or 50CH (bioMérieux), PCR or MALDI TOF MS (Morka et al., 2018; Laporte et al., 2015). Furthermore, the determination of serotypes for enteropathogenic Yersinia species can be achieved using serotype-specific antisera. However, this technique is available only in specialized laboratories (Laporte et al., 2015).

The O-specific polysaccharide (also known as O-antigen) is used for the serological characterization of Ye strains. O-antigen is the outermost structure of lipopolysaccharide (LPS) that is essential for the efficient colonization and invasion of the pathogenic strains (Fàbrega and Vila, 2012; Al-Hendy et al., 1992; Kenyon et al., 2016). The O-antigen is linked via the core oligosaccharide to the lipid A of LPS. The O-antigen is a virulence factor for many bacteria (Al-Hendy et al., 1992). LPS of Ye serotype O:3 has a unique structure in which the outer core (OC) forms a branch. The lipid A moiety is abridged via 3-deoxy-d-manno-2-octulopyranosonic acid (Kdo) to the inner core heptose residues onto which either the OC or the O-antigen is linked (Skurnik et al., 1999; Pinta et al., 2010). OC is important for the resistance of YeO:3 to cationic antimicrobial peptides, and it functions as the receptor of bacteriophage φR1-37 (Pinta et al., 2010; Leskinen et al., 2016; Leon-Velarde et al., 2019). The O-antigen of YeO:3, a homopolymer of 6-deoxy-l-altrose moieties, on the other hand, functions as the receptor of phage φYeO3-12 (Leon-Velarde et al., 2019).

Our previous results suggested that tail fiber protein TFP-Gp17 belonging to φYeO3-12 phage tail fiber contained a chaperone domain on the C-terminus of its polypeptide chain and the adhesion domain on its N-terminus end. The 3D structure of this protein was predicted using the Swiss-Model server suggesting to adopt the tertiary structure as adhesin A (PDB code: 3d9x) (Pyra et al., 2020). That might suggest the adhesive feature for this protein. In this paper, we characterize the ability of bacteria-specific binding by the TFP-Gp17. The TFP-Gp17 binds to the O-antigen of YeO:3 and can be applied to identify strains of this pathogenic serotype. As no other serotype was recognized by TFP-Gp17, the specific detection of YeO:3 bacteria with the help of TFP-Gp17 becomes fast and effective.

## Materials and methods

### Gene cloning, protein overexpression, purification and analysis

The annotated nucleotide sequence of the phage φYeO3-12 genome is available at GenBank under the accession number AJ251805 (Pajunen et al., 2000). The phage genomic DNA was isolated from the phage lysate using a viral DNA extraction kit (Biocompare) and used as a template (20 ng) in the PCR reaction. The TFP-Gp17 encoding gene, g17, was amplified as a 1937 bp fragment by PCR using primers: TFPgp17_FW 5’-TACTTCCAATCCAATGCCATGGCTACAACTATTAAGACCG and TFPgp17_RV 5’- TTATCCACTTCCAATGTTACTAAGTCTTGTCCTTCTCCAAC. The ligation-independent cloning method was used to clone the PCR fragment into the pMCSG9 vector using a T4 DNA polymerase (Eschenfeldt et al., 2009). This way the protein coding sequence would be fused to hexa-His tag—MBP (Maltose Binding Protein). MBP is one of the most popular fusion components for recombinant proteins produced in a bacterial expressing system. MBP facilitates the proper folding and solubility of the target proteins, increasing the effectivity of proteins production (Lebendiker and Danieli, 2010). The construct was transformed into *E. coli* DH5α cells using the heat-shock method, and confirmed by sequencing. The obtained plasmid pMCSG9-6HMBP-ypQ9T0Z9 was transformed into competent *E. coli* BL21(DE3)pLysS.

The *E.coli* BL21(DE3)pLysS/pMCSG9-6HMBP-ypQ9T0Z9 bacteria were inoculated into Luria–Bertani (LB) medium supplemented with ampicillin and chloramphenicol, at 100 and 25 µg/ml, respectively. The bacteria were grown at 37 °C with shaking 120 rpm to an OD600 of 0.7 and the gene expression was induced by addition of isopropyl β-d-thiogalactopyranoside (IPTG) to a final concentration of 0.4 mM, and the bacteria were then incubated overnight at 18 °C. The bacteria were harvested by centrifugation (5000*g*, 5 min) and sonicated 10 times (30-s pulses separated by 15-s breaks) in buffer containing 20 mM Tris/HCl buffer, pH 8.0, 300 mM NaCl, 5% glycerol and 5 mM β-mercaptoethanol (buffer A). The cell disruption by sonication was performed on ice using a UP200S ultrasonic disintegrator (Dr. Hielscher GmbH). The cell debris was pelleted and the supernatant was treated with viscolase (AA&Biotechnology) to reduce the viscosity of the bacterial lysate and filtered through a 0.45 µm filter. Then supernatant was loaded onto Super Nickel NTA Affinity Resin (Protein Ark) equilibrated with buffer A. TFP-Gp17 was purified using two rounds of nickel-affinity chromatography. Unbound proteins were washed with buffer A and TFP-Gp17 was eluted with buffer A containing 250 mM imidazole. Before the second round, the eluted protein fraction was precipitated with 0,65 g/ml ammonium sulfate and harvested by centrifugation (20,000*g*, 4 °C, 45 min) and resuspended in buffer A. To remove the MBP and the his-tag the protein solution was digested by TEV protease overnight at 4 °C. After digestion, the tag-free protein appeared in the unbound fraction of proteins (flow through) during nickel-affinity chromatography. The flow through fraction was again precipitated by addition of 0,65 g/ml ammonium sulfate overnight in 4 °C and harvested by centrifugation (20,000*g*, 4 °C, 45 min). The Knauer system of chromatography was used for protein purification by affinity chromatography. The system was controlled using the Purity Chrome software.

Size Exclusion Chromatography (SEC) was performed using the Dionex Ultimate 3000 System (Dionex Corporation, USA) equipped with LPG-3400SD pump, WPS-3000T(B) FC Analytical autosampler, TCC-3000SD column compartment and a DAD-3000 diode array detector. Protein separation was performed using Superdex®200 10/300 GL Code No 17-5175-01 Id No 0710071 (GE Healthcare). Nominal separation range for this column is 10–600 kDa (globular proteins). A PBS buffer (pH 7.2) was used as the mobile phase. The flow rate was 0.9 ml/minute and the eluent was monitored at 220 nm at room temperature. The inject sample volume was 200 µl. Control of the system, and data acquisition and treatment were performed using Chromeleon software (Dionex).

A Dionex 3000 RS-HPLC equipped with a DGP-3600 pump, a WPS-3000 TLS TRS autosampler, a TCC-3000 RS column compartment (Dionex Corporation, USA) and a Bruker micrOTOF-QII mass spectrometry as a detector (Bruker Daltonics, Germany) were used to determine the molecular weight (MW) of TFP-Gp17. The chromatography column was a 100 × 1 (i.d)-millimeter Thermo Scientific BioBasic-8 with 5-micron particles (Part No. 72205-101030, Serial No. 10158875). The injected sample volume was 2 µl. The flow rate was 0.1 ml/minute and the eluent was monitored using mass spectrometry. The mobile phase: solvent A—0.1% formic acid in water and solvent B—0.1% formic acid in acetonitrile. The ramp: 0 min—5%B, 1 min—5% B, 16 min—95% B, 17 min—95% B, 17.1 min—5% B. The mass spectrometer was calibrated at the beginning of each run with 10 mM sodium formate and the following settings in positive ESI mode were used. Scan range: 300–3000 m/z, End plate offset: − 500 V, Capillary: − 4000 V, Nebulizer gas (N2): 1 bar, Dry gas (N2): 8 L/min, Dry Temperature: 180 °C.

The protein sample was analyzed via 12% SDA-PAGE (Laemmli, 1970) and the concentration was determined by the nanodrop at 280 nm absorbance (Denovix) and by the BCA method (Smith et al., 1985).

### Bacterial cell-based sandwich ELISA

For the bacterial cell-based sandwich ELISA the protocol by Paton et al. (2001) with some modification was used. The bacterial strains used for the experiment are shown in Table 1. Firstly, the bacterial culture was incubated overnight (O/N). Next day, bacterial cultures were refreshed and incubated at 37 °C and 28 °C with shaking 120 rpm until the OD600 reached 0.5. When the OD600 reached the appropriate value, the cultures were washed with PBS-T (PBS supplemented with 0,1% Tween 20) by centrifugation (2000×*g*, 5 min) to get rid of the medium. The bacterial pellet was resuspended in fresh PBS and diluted tenfold. The bacterial suspension was added to Maxisorp 96-well microplates for overnight at 4 °C. After O/N incubation, plate was centrifuged (600×*g*, 4 °C, 20 min). The plate was fixed with 0.1% glutaraldehyde for 30 min in room temperatures. Then solution was removed by pipetting. The plate was then treated with a solution of 0.1% BSA in PBS supplemented with 0.1 M glycine for 2 h at RT. The plates were blocked with 4% (w/v) BSA (BSA Blocker, Thermo Scientific) for 2 h at RT. After washing with PBS-T, the plate was incubated with protein H/MTFP-Gp17 (8.5 µg/ml diluted in PBS) for 2 h at RT. After washing, HRP-conjugated IgG anti-HisTag monoclonal antibody (Biorad), diluted 1:200 in blocking buffer was added to the wells, and the plate was incubated with antibody for 60 min at 37 °C. After washing, the plate was incubated with TMB substrate (Thermo Scientific) for 5–15 min at RT. Reaction was stopped with 0.18 M sulphuric acid. Color development was measured at 450 nm on a microplate reader (Biotek).

**Table 1:** Strains used to perform ELISA assay

No	Bacterial strain	Serotype	Source
1	*Yersinia enterocolitica* 6471/76-c	O:3	Skurnik, 1984
2	*Yersinia enterocolitica YeO3-R1* (spontaneous rough derivative of 6471/76-c)		Al-Hendy et al., 1992
3	*Yersinia enterocolitica* DSMZ 23,249	O:8	DSMZ-German Collection of Microorganisms and Cell Cultures GmbH
4	*Yersinia enterocolitica* PCM 1879	4/O:3	Polish Collection of Microorganisms PAS (PCM)
5	*Yersinia enterocolitica* PCM 1880	5/O:3	PCM
6	*Yersinia enterocolitica* PCM 1881	O:3	PCM
7	*Yersinia enterocolitica* PCM 2072	O:1	PCM
8	*Yersinia enterocolitica* PCM 2080	O:8	PCM
9	*Yersinia enterocolitica* PCM 2081	O:9	PCM
10	*Yersinia enterocolitica* PCM 1883	1A/O:5A	PCM
11	*Yersinia enterocolitica* PCM 1884	2/O:8	PCM
12	*Yersinia enterocolitica* PCM 1886	O:7,8	PCM
13	*Yersinia enterocolitica* PCM 2090	O:27	PCM
14	*Escherichia coli* PCM 2337	–	PCM
15	*Escherichia coli* B PCM 1630	–	PCM
16	*Pseudomonas aeruginosa* PCM 499	–	PCM
17	*Enterobacter aerogenes* PCM 532	–	PCM

### Immunogold labelling—visualization of the interaction of the phage tail fiber protein with Ye:O3 surface using transmission electron microscopy (TEM)

YeO:3 was grown for 24 h at 28ºC, after this time the bacteria were multiplied by transferring to fresh LB medium and incubated until the culture reached OD600 of 0.4–0.5. The culture was centrifuged (3000×*g*, 10 min), the pellet was washed with PBS and centrifuged again. The preparation was applied to a 200-mesh nickel grid with carbon film for 15 min. Next the excess liquid was sucked away, the H/MTFP-Gp17 protein at concentration of 1 mg/ml was applied to the grid for 30 min. Then the monoclonal anti-Maltose Binding Protein (MBP) antibody (produced in mouse, SIGMA) at a concentration of 1 mg/ml was applied to the grid for 30 min and in the next stage the anti-mouse IgG1 conjugated with HRP (produced in rabbit, SIGMA) at a concentration of 1 mg/ml was applied to the grid for 30 min. The grid was rinsed with PBS buffer supplemented with BSA and the Protein A-Gold with 20 nm colloidal gold was added for 30 min. After this time, the grid was washed with PBS and Milli Q water and contrasted with 2% uranyl acetate. Samples were visualized using a JEOL JEM-1200 EX 80kV TEM.

## Results

### Gene cloning, protein overexpression, purification and analysis

The gene g17 was cloned to the expression vector pMCSG9 in order to obtain both a tagged and untagged variant of the adhesin TFP-Gp17. The tagged variant carried the MBP-His tag to be used as a target for the anti-His and anti-MBP antibodies.

**Fig. 1:**
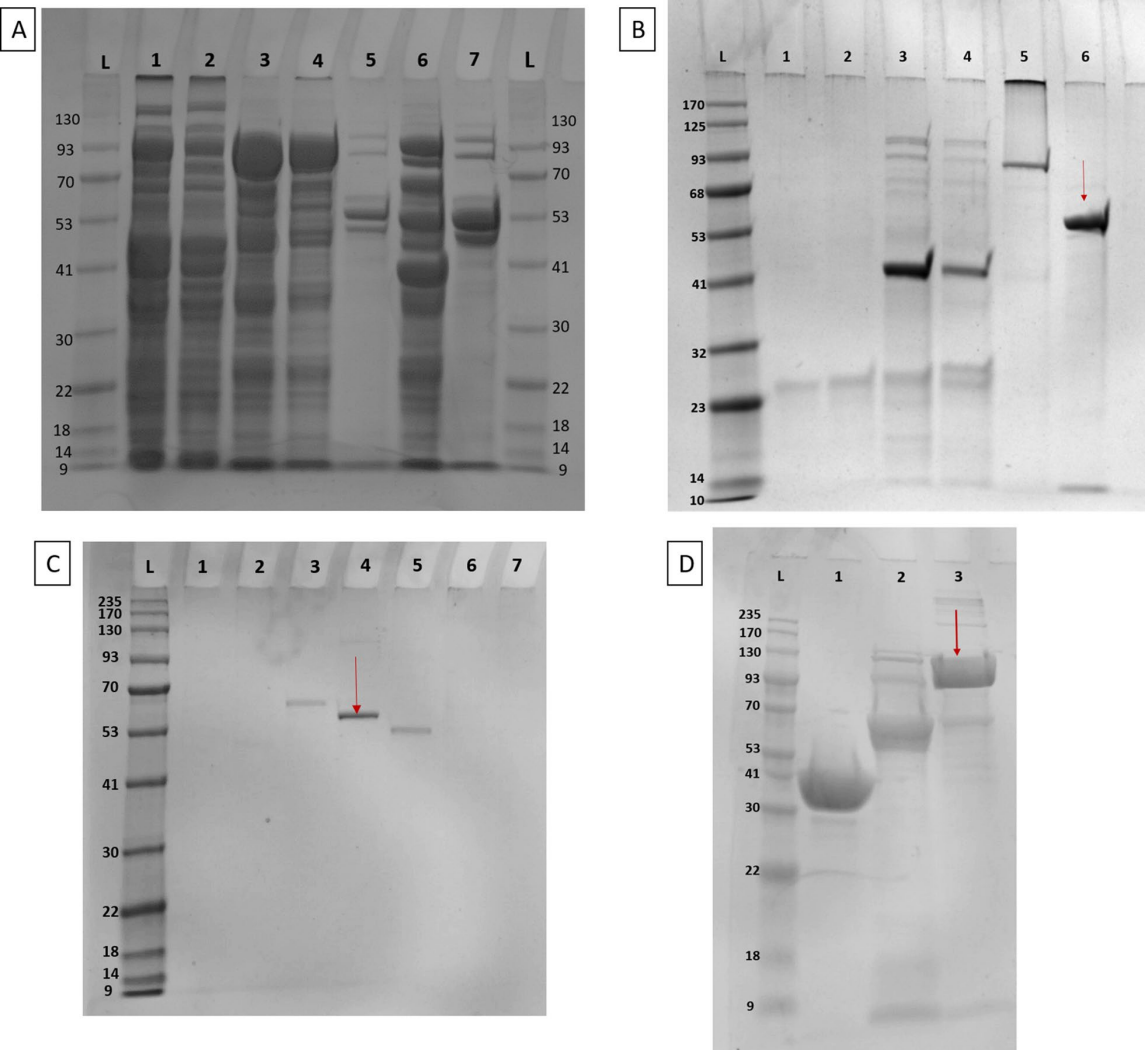
SDS-PAGE of TFP-Gp17. **A** Samples from all purification steps by nickel-affinity chromatography. Lanes: 1, supernatant from bacterial lysate; 2, flow through 1 (FT1) containing the unbound proteins; 3, elution fraction 1 (elution with buffer A supplemented with 250 mM imidazole); 4, elution fraction after desalting with ammonium sulfate; 5,7, FT2, containing the unbound proteins after cleavage with the TEV protease; 6, elution fraction 2. **B** TFP-Gp17 before SEC chromatography is shown in the lane 6. **C** Protein fractions after SEC chromatography; TFP-Gp17 in 4 lane is indicated by the red arrow. **D** The H/MTFP-Gp17 protein after SEC chromatography is indicated in lane by the red arrow

The overexpressed TFP-Gp17 carrying the MBP-His tag (H/MTFP-Gp17) was purified using two rounds of nickel-affinity chromatography followed by SEC as described in the Materials and Methods section. The recovered protein was analyzed in SDS-PAGE (Fig. 1).

Based on these analyses, the purity of both TFP-Gp17 and H/MTFP-Gp17 was over 95%. After digestion of TFP-Gp17 with TEV protease two polypeptides were identified in mass analysis with molecular masses after deconvolution of 57.423 and 12.242 kDa. Their combined mass of 69.665 kDa corresponds well with the predicted mass of 69.4 kDa of TFP-Gp17 before autoproteolysis. The mass spectrometry indicated that the molecular mass of H/MTFP-Gp17 was 100.846 kDa, and that also another protein with a molecular mass of 12.242 kDa was present. Their combined mass of 113.088 kDa corresponds to the predicted mass of H/MTFP-Gp17 before autoproteolysis (Additional file 1: Figures S1 and S2). The autoproteolysis indicates that the C-terminal fragment of TFP-Gp17 is cleaved, supporting our previous observation that TFP-Gp17 contains an S74 peptidase domain, which is responsible for protein autolysis (Pyra et al., 2020). Importantly, the results also demonstrated that there are no sterical obstacles for the autoproteolysis in the H/MTFP-Gp17 construct where the His-MBP is fused to the N-terminal end of TFP-Gp17.

### Bacterial cell-based sandwich ELISA

**Fig. 2:**
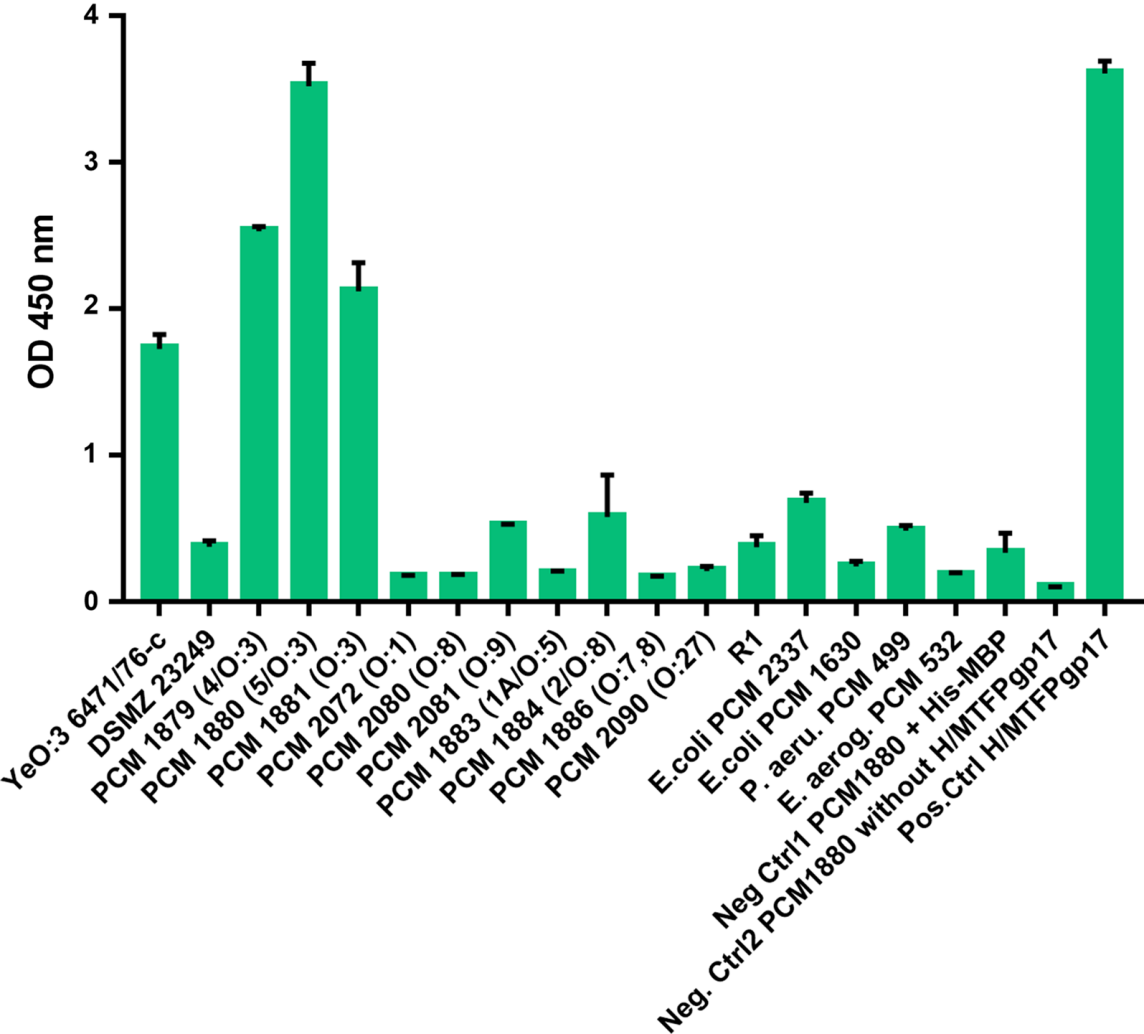
H/MTFP-Gp17—based ELISA for specific detection of YeO:3 (10^5^ CFU/ml). The bound H/MTFP-Gp17 was detected by HRP-conjugated IgG anti-HisTag monoclonal antibody. The color development was measured at 450 nm on a microplate reader. SD was calculated from 3 replicates

We used an ELISA-based method for the specific detection of the pathogenic YeO:3 bacteria employing H/MTFP-Gp17 as the recognition agent. Microtiter plate wells were coated with whole bacteria and the bound H/MTFP-Gp17 was detected by the HRP-conjugated anti-His antibodies. The specificity of the assay was tested against pathogenic serotype O:3, O:8, O:9, and O:5 strains responsible for Yersiniosis in Europe, and against several other non-Ye pathogens. We also included the rough YeO:3 mutant strain YeO3-R1 devoid missing the O-antigen. As negative controls we used samples that contained the YeO:3 bacteria without H/MTFP-Gp17 or with His-MBP (Fig. 2), while H/MTFP-Gp17 alone served as positive control for the protein interaction with anty-HisTag antibody. The experiment was repeated three times and each time the signal from O:3 strains was the highest.

Among the four YeO:3 strains, the biotype 5 strain had the highest signal with the absorbance at 450 nm of over 3.5. The absorbances of the other O:3 strains were between 1.5 and 2.5. The O-antigen negative mutant YeO3-R1 was not recognized by the phage adhesion (The absorbance was below 0.5). Among the non-Ye:O3 strain, *E coli* PCM2337 gave the highest signal, ~ 0.7, at the same test conditions. In the test conditions, a signal higher than 1.0 at 450 nm may be a safe cut-off above which the detected strain certainly belongs to the YeO:3 serotype. These results indicated that TFP-Gp17 is responsible for the recognition of the bacteria and that the O-antigen of YeO:3 bacteria is the receptor. The results also clearly show that MBP does not bind non-specifically to bacteria.

**Fig. 3:**
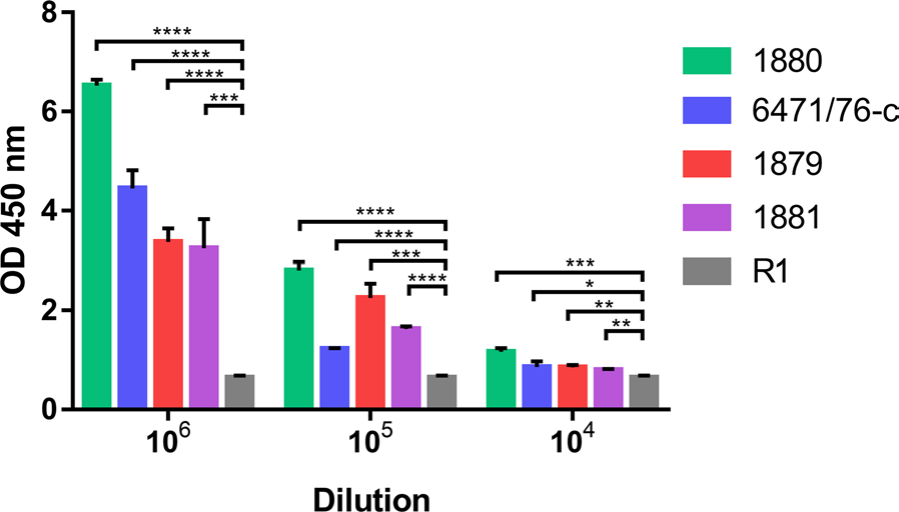
The sensitivity the H/MTFP-Gp17—based ELISA for specific detection of YeO:3 bacteria. The wells were coated with YeO:3 bacteria at indicated concentrations. For the detection H/MTFP-Gp17 at 8.5 µg/ml was used, and the bound H/MTFP-Gp17 was detected as described in Fig. 4 legend. As the negative control R1 strain was used. SD was calculated from 3 replicates. T-test was performed between tested strains and control strain R1 (*- P ≤ 0.05; **- P ≤ 0.01; ***- P ≤ 0.001; ****- P ≤ 0.0001)

The sensitivity of the ELISA was assessed by coating the wells with final bacterial concentrations of 10^6^, 10^5^ and 10^4^ CFU/ml of the four YeO:3 strains (Fig. 3).

Taking into account the safe cut-off value (estimated as 1.0), the limit of bacteria detection in this ELISA conditions may be determined as not less than 10^5^ CFU.

### Visualization of the phage adhesin interaction with YeO:3 using TEM

**Fig. 4:**
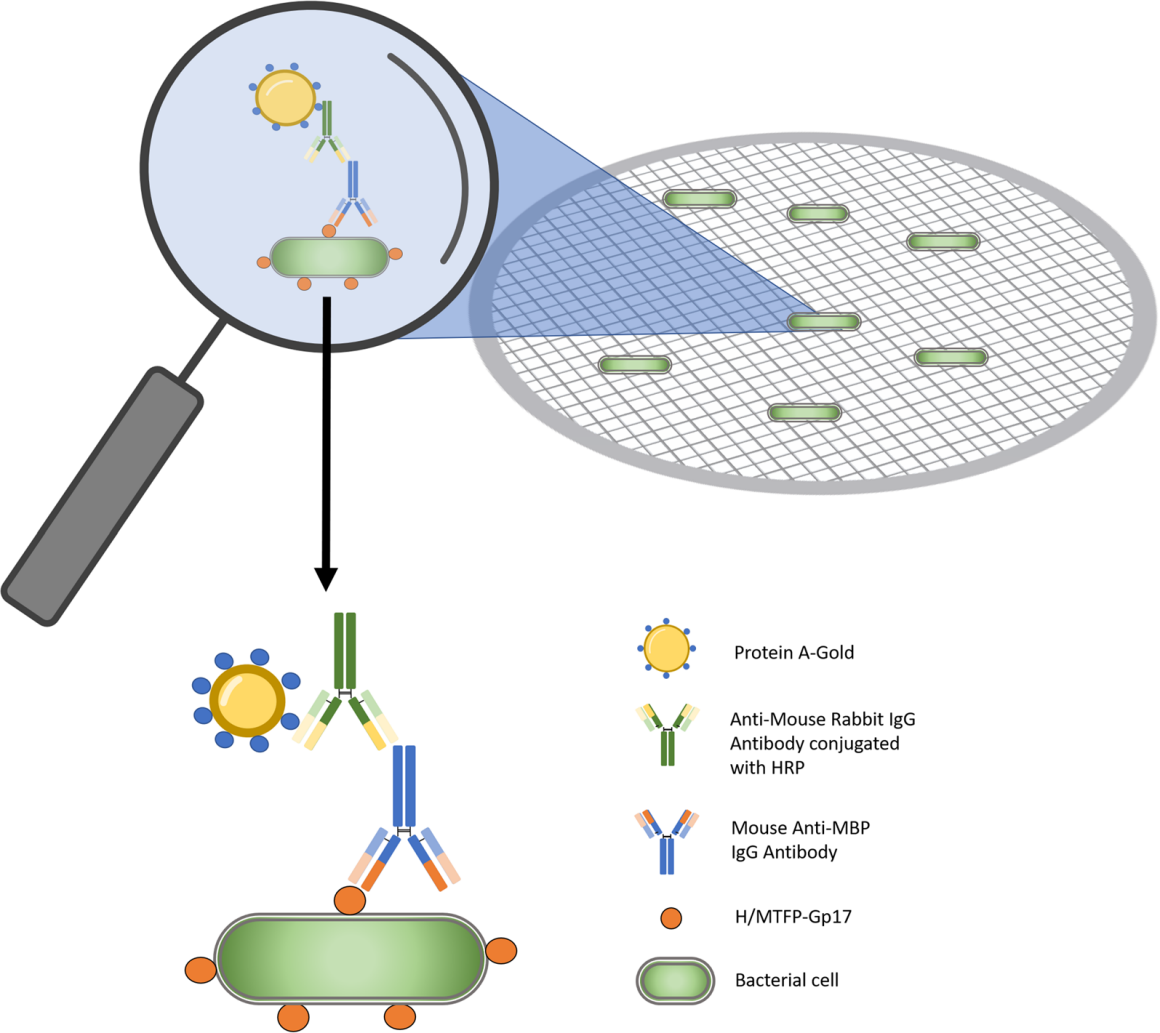
Sandwich-type preparation method based on immunogold labelling to visualize by TEM the interaction of H/MTFP-Gp17 with bacterial cell surface

For the visualization of the interaction between phage protein and bacterial cell, we designed a sandwich-type preparation method based on immunogold labelling according to the scheme represented below (Fig. 4).

**Fig. 5:**
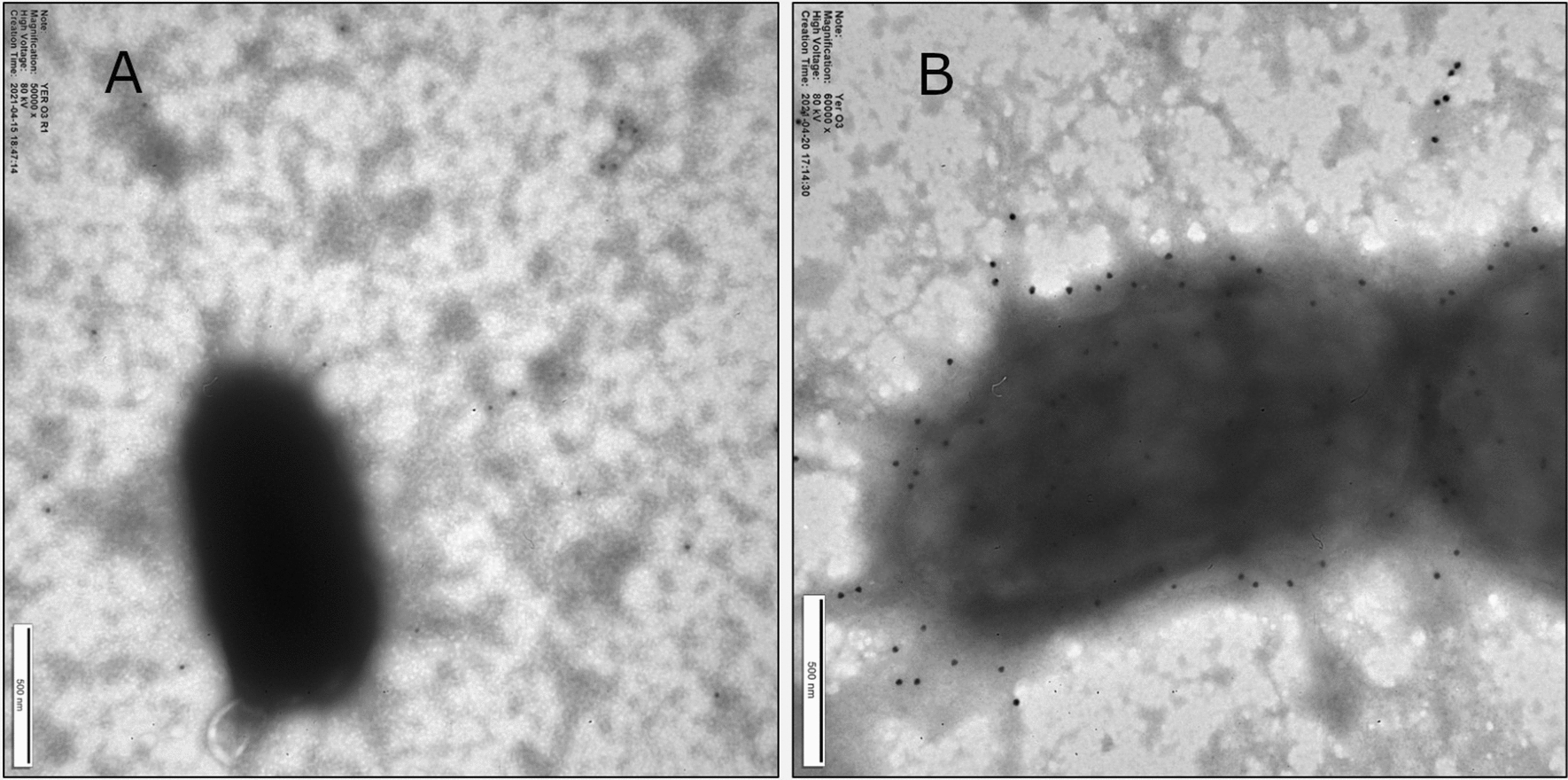
Specific interaction of H/MTFP-Gp17 with YeO:3 bacteria visualized by TEM using immunogold labelling. **A** R1 mutant, the negative reaction with Protein A-Gold, **B** YeO:3 wild type strain 6471/76-c, positive reaction with Protein A-Gold

As a negative control, we used YeO3-R1, the rough derivative of the YeO:3 wild type strain 6471/76-c. The immunogold labelling for both types of bacteria was performed identically. In Fig. 5B gold nanoparticles can be observed on the YeO:3 surface, which indicates a reaction with the antibody conjugated with protein on the bacteria surface We didn’t observe such effect in the case of R1 mutant (Fig. 5A). This is another evidence showing the specific and selective interaction of the protein with bacteria YeO:3 surface (Additional file 1: Figure S3).

## Discussion

Yersiniosis is somewhat underestimated threat to human health. In some countries, *Yersinia* infections have overtaken *Shigella* and *Salmonella* species as the most common cause of bacterial gastroenteritis. Diagnosis towards Ye infections is infrequently performed routinely in clinical laboratories because of Ye specific growth characteristics, which make it difficult to isolate and culture (Aziz and Yalamanchili, 2021). Moreover, current isolation procedures are time-consuming and expensive, thus leading to underestimates of the incidence of enteric yersiniosis, inappropriate prescriptions of antibiotic treatments, and unnecessary appendectomies (Laporte et al., 2015; Weagant and Feng, 2017). Since the Ye serotype O:3 is the most common pathogenic serotype encountered in Europe, we have demonstrated for the first time the ability of the phage φYeO3-12 receptor-binding-protein to interact with the serotype O:3 bacteria in the highly sensitive ELISA. The TFP-Gp17 is most specific towards both pathogenic biotype 5 and 4 of Ye with O:3 serotype (Leon-Velarde et al., 2014). The differences between these two biotypes are determined by presence of some genes responsible for biological activity (Morka et al., 2018). The ability of some tail fiber phage adhesins for bacteria detection was demonstrated previously by Leon-Velarde et al. (2019). The example was RBP Gp17 derived from the Podovirus phage vB_YenP_AP5. The protein was identified as a ligand with specificity also for the O-antigen of serotype O:3 strains. These two adhesins, TFP-Gp17 of φYeO3-12 and RBPgp17 of phage vB_YenP_AP5 are 89% identical with highest similarity at the N-terminal parts of these proteins (Leon-Velarde et al., 2014). Another example was a distal long tail fiber protein, RBP Gp37, derived from the Myovirus phage vB_YenM_TG1, however, it was identified as a ligand for the outer membrane protein OmpF of serotype O:3, O:5,27 and O:9 (Leon-Velarde, 2017). In the case of TFP-Gp17, the specificity is strictly restricted to the serotype O:3.

Our results correspond to those reported by Laporte et al. (2015) where EIA (Enzymatic Immunoassay) was presented for fast Ye detection. Although, Laporte used the monoclonal antibodies in that assay the detection limits are the same also for TFP-Gp17. The advantage of using the phage protein is due to the fact, the procedure of phage adhesin production and purification is faster and easier than monoclonal antibody generation. TFP-Gp17 is produced in complex with MBP. The His-MPB is not cleaved out from the complex since its presence is necessary for the phage adhesin detection after bacteria binding.

To visualize and confirm the interaction of the phage adhesin with whole bacteria we used immunogold labeling method based on the workflow from ELISA with slight modifications. As a negative control, YeO3-R1 mutant was used (Kaur et al., 2002). This experiment confirmed the previous results, gold nanoparticles were abundantly present on the YeO:3 surface in contrast to the YeO3-R1 mutant, which indicates the high specificity of the complex.

In our previous report, we indicated that two amino sugars GalNAc and GlcNAc stabilize the phage adhesin having impact on increase of its thermal stability (Pyra et al., 2020). Initially we thought that this could be due to interaction with the aminosugar moieties presented in the outer core (OC) of YeO:3 (Al-Hendy et al., 1992). For that reason, we took the YeO3-R1 mutant with exposed OC missing the O-antigen to check the interaction. Neither H/MTFP-Gp17 nor TFP-Gp17 bound to YeO3-R1 bacteria in opposition to the wild type YeO:3 bacteria. We decided to assess whether the interaction occurs with those two aminosugars using modified AuNP and UV–Vis spectroscopy (Additional file 1: Figure S4). As the LSPE band is sensitive to the nanoparticles properties, UV–Vis spectroscopy was used to monitor the LSPR band of AuNPgalSH30 and AuNPglukSH30 before and after incubation with the phage adhesin. According to the obtained results, the interaction between the phage adhesin and these two aminosugars took place. We assume that the interaction stabilizes not only the TFP-Gp17 but also the whole phage in the gastrointestinal tract of both humans and pigs since GalNAc as well as GlcNAc are released by enzymes coming from the commensal bacteria (Sicard et al., 2017). As it was previously mentioned, these aminosugars are also a carbon source for Ye strains (Schmühl et al., 2019). Moreover, Ye contain mucin-degrading enzyme(s) increasing the permeability of the mucus gel layer and allowing the bacteria to move more easily through the mucin (Sicard et al., 2017).

For the first time, the TFP-Gp17 of phage φYeO3-12 was demonstrated as a highly specific adhesin towards the pathogenic Ye serotype O:3. It was shown that TFP-Gp17 carrying the 6His-MBP tag could be used as a sensing molecule to detect the YeO:3 strains. The phage adhesin recognizes the serotype O:3 O-antigen of the bacterial LPS. The YeO3-R1 mutant strain lacking the O-antigen was not recognized by the phage protein. Both the specificity and sensitivity of the ELISA test were determined. We also showed that the phage receptor binding adhesin protein interacts with the two major mucus components GalNAc and GlcNAc which suggests that the amino sugars stabilize the whole phage particle in the intestinal tracts of mammals.

## Supplementary Information

**Additional file 1:** Additional file contains partial implementation, Figures S1–S4.

## Data Availability

All data generated or analysed during this study are included in this published article (and its additional files).
