# Supplementary material for: φYeO3-12 phage tail fiber Gp17 as a promising high specific tool for recognition of Yersinia enterocolitica pathogenic serotype O:3
Source: AMB Express. 2022 Jan 6;12:1. doi: 10.1186/s13568-021-01341-2 (PMC8739404; doi:10.1186/s13568-021-01341-2)
Supplement: Supplementary file 1 — Additional file 1: Additional file contains partial implementation, Figures S1–S4. [file 13568_2021_1341_MOESM1_ESM.docx]

*Additional file 1.*

AMB Express

**φYeO3-12 phage tail fiber Gp17 as a promising high specific tool for recognition of *Yersinia enterocolitica* pathogenic serotype O:3**

Karolina Filik^1^, Bożena Szermer-Olearnik^1^, Joanna Niedziółka-Jönson^2^, Ewa Roźniecka^2^, Jarosław Ciekot^1^, Anna Pyra^3^, Irwin Matyjaszczyk^4^, Mikael Skurnik^5^,^6^ and Ewa Brzozowska^1*^

1. Hirszfeld Institute of Immunology and Experimental Therapy, Polish Academy of Sciences, 12 R. Weigl St, 53114,Wroclaw, Poland
2. Institute of Physical Chemistry, Polish Academy of Sciences, Kasprzaka 44/52 01-224 Warsaw, Poland
3. University of Wroclaw, Faculty of Chemistry, 14 F. Joliot-Curie St, Wroclaw, 50383, Poland

4. Department of Mycology and Genetics, Institute of Genetics and Microbiology, University of Wrocław, 51-148, Poland
5. Department of Bacteriology and Immunology, Human Microbiome Research Program, Faculty of Medicine, University of Helsinki, Helsinki, Finland
6. Division of Clinical Microbiology, Helsinki University Hospital, HUSLAB, Helsinki, Finland

*Correspondence: ewa.brzozowska@hirszfeld.pl;

Tel.: +48 71 370 99 01, Fax: +48 71 337 13 82

**Results:**

***Mass spectra***


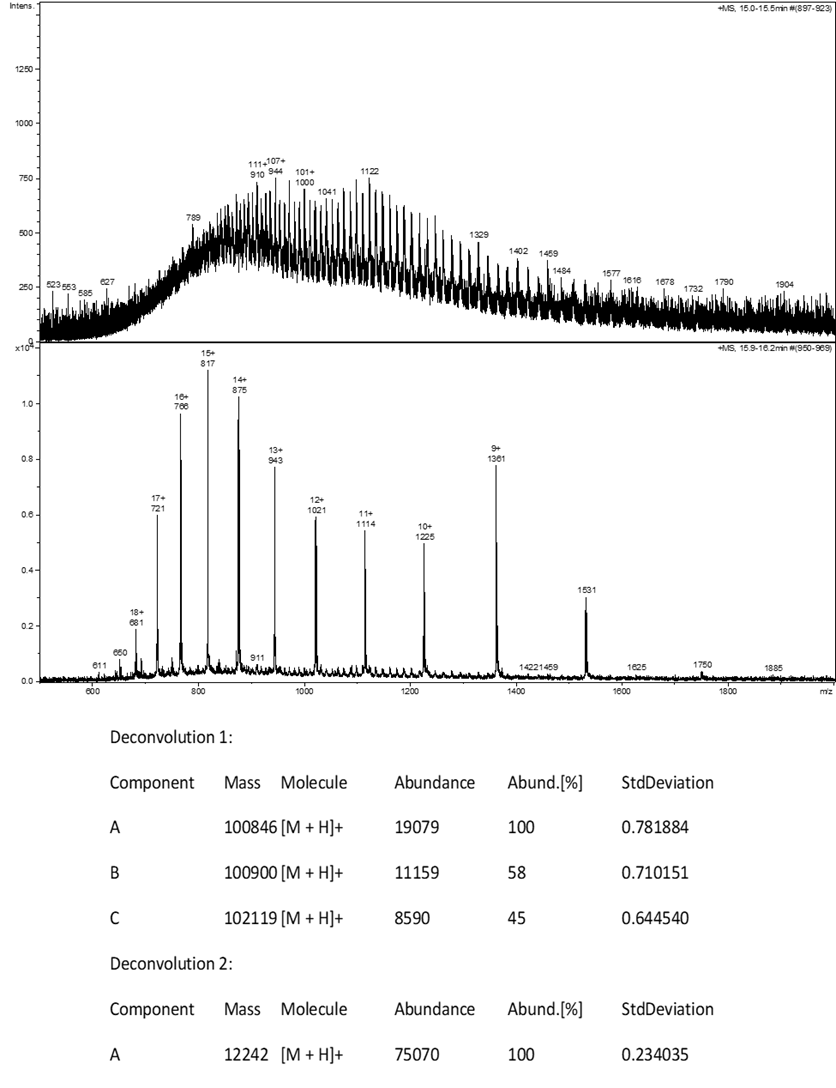
Figure 1S. Mass spectra of H/MTFP-Gp17 complex


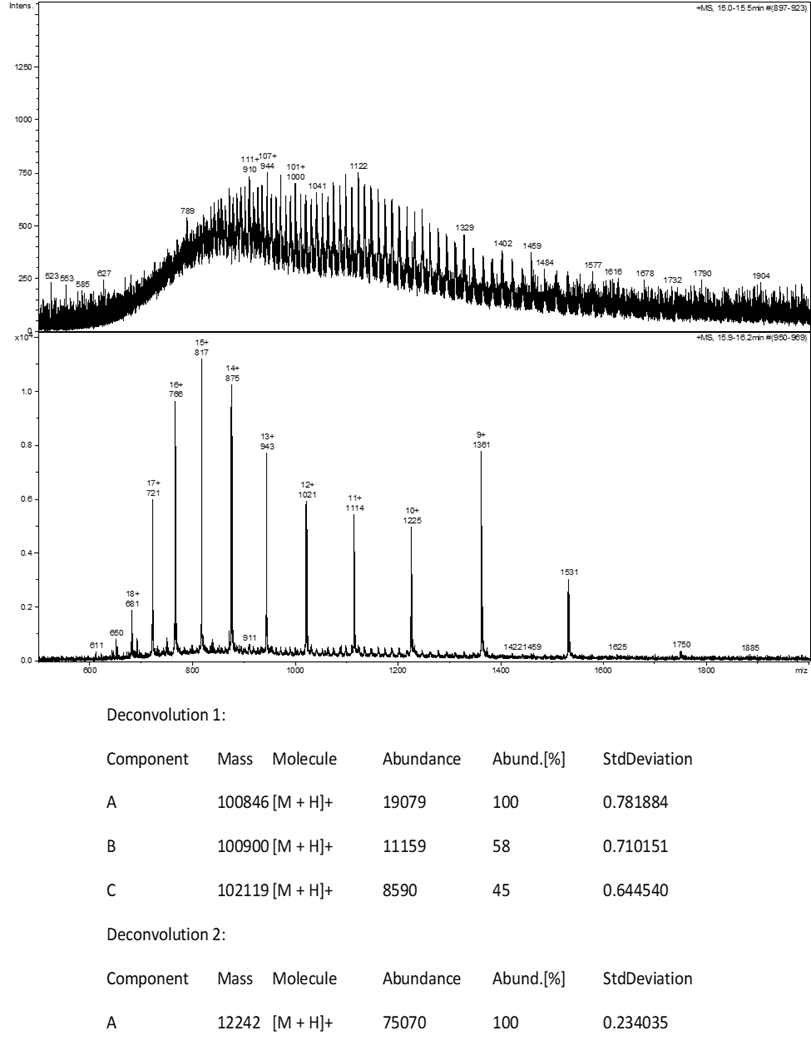


Figure 2S. Mass spectra of TFP-Gp17 after digestion with TEV protease


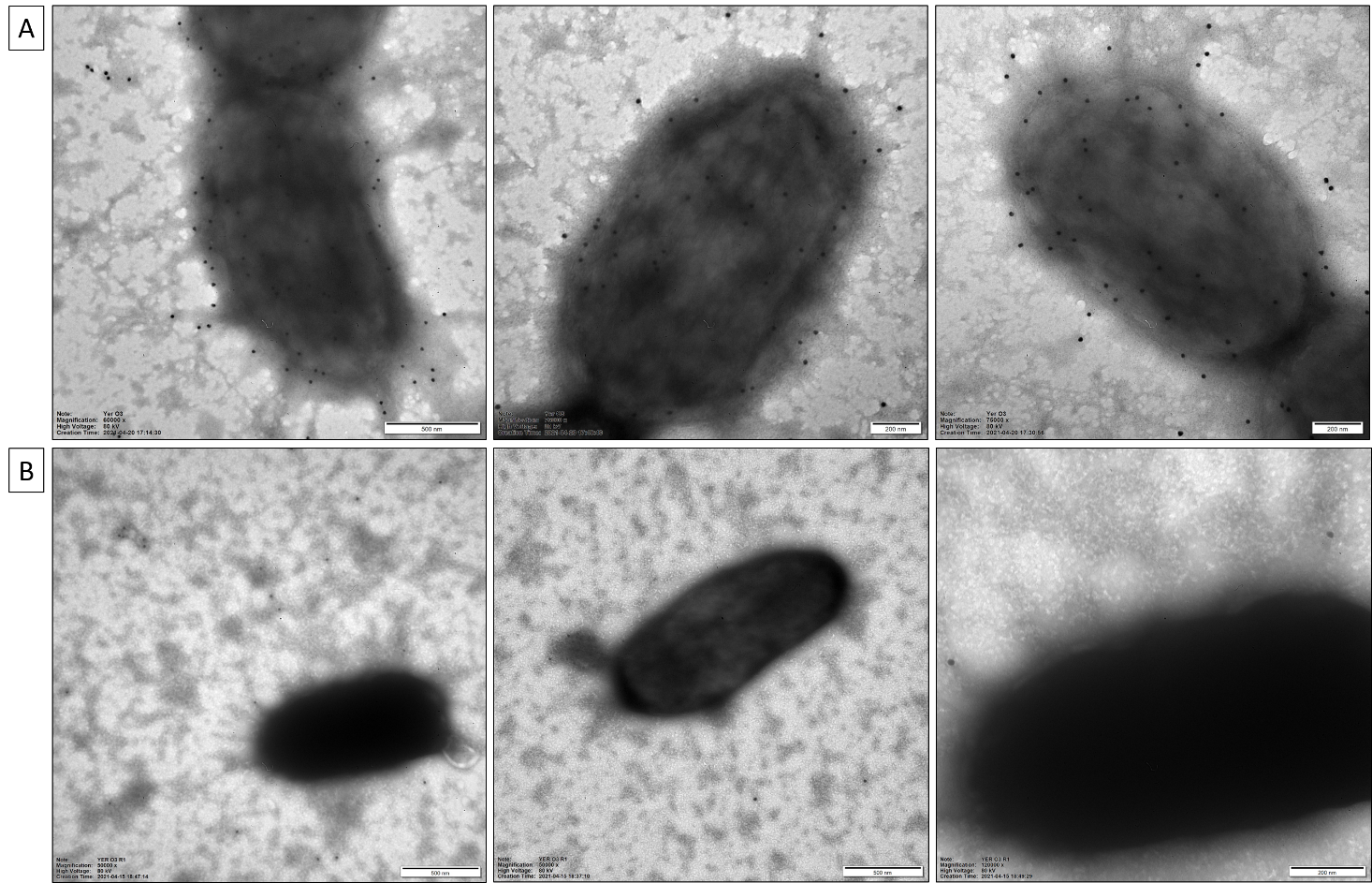


***Visualization of the phage adhesin interaction with YeO:3 using TEM***

Figure 3S. Specific interaction of H/MTFP-Gp17 with YeO:3 bacteria visualized by TEM using immunogold labelling. (A) YeO:3 wild type strain 6471/76-c, positive reaction with Protein A-Gold, (B) R1 mutant, the negative reaction with Protein A-Gold.

***Material and Methods for Assessment of binding between TFP-Gp17 and gold nanoparticles coated by thiol derivatives 218 of GalNAc and GlcNAc 219***

Synthesis of gold nanoparticles (AuNP) stabilized with citrates was performed as 220 described before [23] 150 ml of a 0.5 mM HAuCl4 3×H2O solution was heated up to 95° C 221 on a heating plate with intense stirring (900 rpm). Then, 15 ml of 38.8 mM trisodium citrate 222 dihydrate was added. The solution was further heated for 18 min and cooled. Next, 5 ml 223 of AuNP stock suspension and 5 ml of ethanol were added to 2 vials. Then, 30 μl of 2.12 224 μM ethanolic solution of 11-(α-D-galactopyranosyl) undecane-1-thiol (gal-SH) 225 (ProChimina) and 11-(α-D-glucopyranosyl) undecane-1-thiol (gluc-SH) (ProChimina) 226 were added, respectively. The modified nanoparticles were marked as AuNPgalSH30 and 227 AuNPglukSH30. UV-Vis spectroscopy was used to characterize synthesized AuNP and 228 their surface modification, as well as tracking the interaction between the immobilized 229 aminosugars and the adhesin TFP-Gp17, by following the changes of the localized surface 230 plasmon resonance (LSPR) band with a maximum wavelength (λmax) at around 530 nm. 231 All measurements were performed in a BRAND semi-micro cuvette made from PMMA 232 with an optical path of 10 mm. First, the suspensions of AuNP as synthesized and 233 modified with aminosugars were measured with addition of PBS and BSA as reference 234 probes. Finally, TFP-Gp17 in PBS was added to a final concentration of 10 μg/ml and after 235 2 h, the spectra were measured once again.

***Results for Assessment of the binding between TFP-Gp17 and gold nanoparticles modified with thiol de-339 rivatives of GalNAc and GlcNAc 340***





Figure 4S Extinction spectra for AuNPGalNacSH (black) and AuNPGlcNacSH (red) suspensions before and after (green and blue) 2h incubations with 10 μg/ml TFP-Gp17.

In our previous report, we indicated that TFP-Gp17 is stabilized by the aminosugars GalNAc and GlcNAc [1] suggesting an interaction of the adhesin with these sugars. To assess the interaction between the aminosugars and the TFP-Gp17 adhesin we used colorimetric detection [2]. This is possible because the wavelength of the localized surface plasmon resonance of the AuNPs is strongly dependent on the dielectric constant of the local environment. Therefore, we synthesized gold nanoparticles with covalently immobilized GalNAc and GlcNAc marked as AuNPGalNacSH and AuNPGlcNacSH, respectively. In the first control experiments PBS and BSA were added to the AuNPGalNacSH and AuNPGlcNacSH suspensions to see if a change of the ionic strength, in the case of PBS, or sugar protein interaction, in the case of BSA, affected the position of the LSPR. No changes in the spectra were observed (data not shown). Then the spectra of AuNPGalNacSH and AuNPGlcNacSH nanoparticles were measured before (black and red color in Figure 1) and after incubation with the TFP-Gp17 (blue and green color) under the same experimental conditions. The results are presented in Figure 4.

The LSPR bands at 531 nm of AuNPGlcNacSH and AuNPGalNacSH after incubation with the adhesin were shifted to 537 nm and 553 nm respectively, and the extinction decreased significantly. This directly indicates an interaction between the phage protein and the GalNAc and GlcNAc coated particles. The shift for GalNAc was more pronounced than that for GlcNAc. This shows that refractive index in the closest vicinity of AuNPGalNacSH changed significantly. Moreover, a second band around 610 nm is observed due to changes of the interparticle distance and aggregates of AuNPGalNacSH formation caused be the complex formation between the aminosugar and adhesin [2,3]. Only a small shoulder is seen in this position in the case of AuNPGlcNacSH.

**Bibliography:**

[1] Matyjewicz, J., Lesniewski, A., & Niedziolka-Jonsson, J. (2014). Click chemistry modification of glassy carbon electrode with gold nanoparticles for electroactive ion discrimination. Electrochemistry communications, 48, 73-76.
doi: 10.1016/j.elecom.2014.08.020

[2] Kannan, P., Los, M., Los, J. M., & Niedziolka-Jonsson, J. (2014). T7 bacteriophage induced changes of gold nanoparticle morphology: biopolymer capped gold nanoparticles as versatile probes for sensitive plasmonic biosensors. Analyst, 139(14), 3563-3571.
doi: 10.1039/C3AN02272B

[3] Rechberger, W., Hohenau, A., Leitner, A., Krenn, J. R., Lamprecht, B., & Aussenegg, F. R. (2003). Optical properties of two interacting gold nanoparticles. Optics communications, 220(1-3), 137-141. doi: 10.1016/S0030-4018(03)01357-9
